# Transcriptome and proteome dynamics in larvae of the barnacle *Balanus Amphitrite* from the Red Sea

**DOI:** 10.1186/s12864-015-2262-1

**Published:** 2015-12-15

**Authors:** Kondethimmanahalli H. Chandramouli, Sarah Al-Aqeel, Taewoo Ryu, Huoming Zhang, Loqmane Seridi, Yanal Ghosheh, Pei-Yuan Qian, Timothy Ravasi

**Affiliations:** KAUST Environmental Epigenetic Program (KEEP), Division of Biological and Environmental Sciences and Engineering, King Abdullah University of Science and Technology, Thuwal, Kingdom of Saudi Arabia; Division of Applied Mathematics and Computer Sciences, King Abdullah University of Science and Technology, Thuwal, Kingdom of Saudi Arabia; Bioscience Core Laboratory, King Abdullah University of Science and Technology, Thuwal, Kingdom of Saudi Arabia; KAUST Global Collaborative Research Program, Division of Life Science, The Hong Kong University of Science and Technology, Hong Kong, Hong Kong

**Keywords:** *B. amphitrite*, Red sea, Transcriptomics, Proteomics, Adaptation

## Abstract

**Background:**

The barnacle *Balanus amphitrite* is widely distributed in marine shallow and tidal waters, and has significant economic and ecological importance. Nauplii, the first larval stage of most crustaceans, are extremely abundant in the marine zooplankton. However, a lack of genome information has hindered elucidation of the molecular mechanisms of development, settlement and survival strategies in extreme marine environments. We sequenced and constructed the genome dataset for nauplii to obtain comprehensive larval genetic information. We also investigated iTRAQ-based protein expression patterns to reveal the molecular basis of nauplii development, and to gain information on larval survival strategies in the Red Sea marine environment.

**Results:**

A nauplii larval transcript dataset, containing 92,117 predicted open reading frames (ORFs), was constructed and used as a reference for the proteome analysis. Genes related to translation, oxidative phosphorylation and cytoskeletal development were highly abundant. We observed remarkable plasticity in the proteome of Red Sea larvae. The proteins associated with development, stress responses and osmoregulation showed the most significant differences between the two larval populations studied. The synergistic overexpression of heat shock and osmoregulatory proteins may facilitate larval survival in intertidal habitats or in extreme environments.

**Conclusions:**

We presented, for the first time, comprehensive transcriptome and proteome datasets for Red Sea nauplii. The datasets provide a foundation for future investigations focused on the survival mechanisms of other crustaceans in extreme marine environments.

**Electronic supplementary material:**

The online version of this article (doi:10.1186/s12864-015-2262-1) contains supplementary material, which is available to authorized users.

## Background

Barnacles are marine dominant fouling organisms accumulate on ship hulls, piers and under water structures; biofouling by these invertebrates results in an increase in power and fuel consumption [1–3] associated with marine transport. Removing biofouling barnacles is a major expense involving significant economic losses in shipping and other marine industries. However, some barnacles are of great commercial importance; for example, the gooseneck barnacle achieves an average market price of 30–60 USD per kilogram, depending on the season [4]. Such species are highly exploited, involving harvesting of approximately 500 kg per season in Spain and Portugal, and hence there is an urgent need for strategies to protect the stocks. The barnacle *Balanus amphitrite* is a widely distributed dominant fouling organism and a major component of rocky shore benthic communities [5–7]. Therefore, *B. amphitrite* is an excellent model organism for research on settlement biology, adaptation and biofouling. Nauplii are the earliest free-swimming stage in the development of most crustaceans, including barnacles [8], and are extremely abundant in the marine ecosystems. Barnacle embryos are brooded by the parents, and the nauplii are released into the water column as swimming larvae [9]. The nauplius I stage has an elongated and pear-shaped body, and the abdominal and caudal spines are partially differentiated. The eye is located in a front medial position and occurs in all developmental stages. The presence of posteriorly folded frontal horns and simple setae on the limbs are characteristics of the nauplius I stage. The newly released larvae swim actively and molt to the next developmental stage (nauplius II) within 30 min [10]. In the nauplius II stage the carapace extends in all directions and the entire body develops a bell shape that has numerous small spines and a pair of prominent spines. The nauplius II molts to the nauplius III stage in 38 h [11]. The nauplius III stage undergoes several moults before it transforms into a cyprid stage [12]. Molecular studies defining the role of gene or protein expression signatures in development and adaptive strategies of marine larvae in the natural marine environment are limited. Previous studies have used a broad range of proteomics and genomic tools to identify proteins and genes in larval stages of barnacles. However, these studies have mainly focused on exploring settlement and attachment processes. For example, Chen et al. (2011) identified 7954 putative differentially-expressed genes in larvae and adults of *B. amphitrite* using 454 pyrosequencing [13], and Yan et al. (2012) carried out *in silico* transcriptome data mining to study neuropeptides and their possible functional role in barnacle larval settlement [14]. Similarly, De Gregoris et al. (2012) generated deep sequencing EST libraries for nauplii and the cyprid and adult stages of *B. amphitrite* and identified unique contigs for each developmental stage [15]. More recently, Lin et al. (2014) performed Illumina sequencing of a membranous-based barnacle and identified cement- and adhesion-related genes [16]. Thiyagarajan and Qian (2008) reported the first study of global proteome expression patterns in cyprids and metamorphosed larvae of *B. amphitrite* [17], and Zhang et al. (2010) identified proteins from cyprid larvae using prefractionation methods to reduce sample complexity [18]*.* Chandramouli et al. (2012) used multiplex proteomics to describe a distinct protein glycosylation pattern during barnacle larval metamorphosis [19], and Chen et al. (2014) recently used label-free quantitative proteomics to identify 700 proteins from nauplii II and VI, cyprids, and juvenile stages of *B. amphitrite* [20]. In addition, several studies have demonstrated the feasibility of using proteomics tools to investigate the mode of action of antifoulant chemicals in preventing barnacle larval settlement [21–23]. These studies have provided a wealth of molecular information, but comprehensive genome and proteome datasets for nauplius larvae are still lacking. Thus, in the present study we revealed sequence information and investigated the dynamics of protein expression patterns of nauplius larvae. Patterns of differential protein expression between two larval populations from contrasting marine environments were screened to investigate the survival strategies of larvae. Extreme marine environments pose great challenges for the survival of marine larvae. For example, the Red Sea (RS) is one of the warmest, most saline bodies of water; it receives little rainfall and has reduced oxygen solubility and high evaporation rates [24, 25]. The ranges of salinity (36–41psu) and temperature (30–33 °C) in the RS are considered extreme. In contrast, in Hong Kong (HK) marine water the salinity and temperature ranges are 28–31psu and 26–28 °C, respectively [26, 27]. The RS has a low concentration of dissolved oxygen (4.3–5.6 mg/L) compared with that of HK marine water (6.1–7.3 mg/L). Therefore, we hypothesized that changes in protein expression patterns would reflect the adaptation of barnacle larvae to the RS marine environment. To test this hypothesis we adopted a population proteomics approach to compare protein expression in larvae from HK marine waters and the RS. The specific objectives were: (i) to generate a comprehensive transcriptome dataset for the nauplius larvae of *B. amphitrite*; (ii) to compare the proteomes of barnacle larvae populations from the RS and HK; and (iii) to explore survival strategies of larvae in the RS environment.

## Results

### Transcriptome of RS barnacle nauplii

**Fig. 1:**
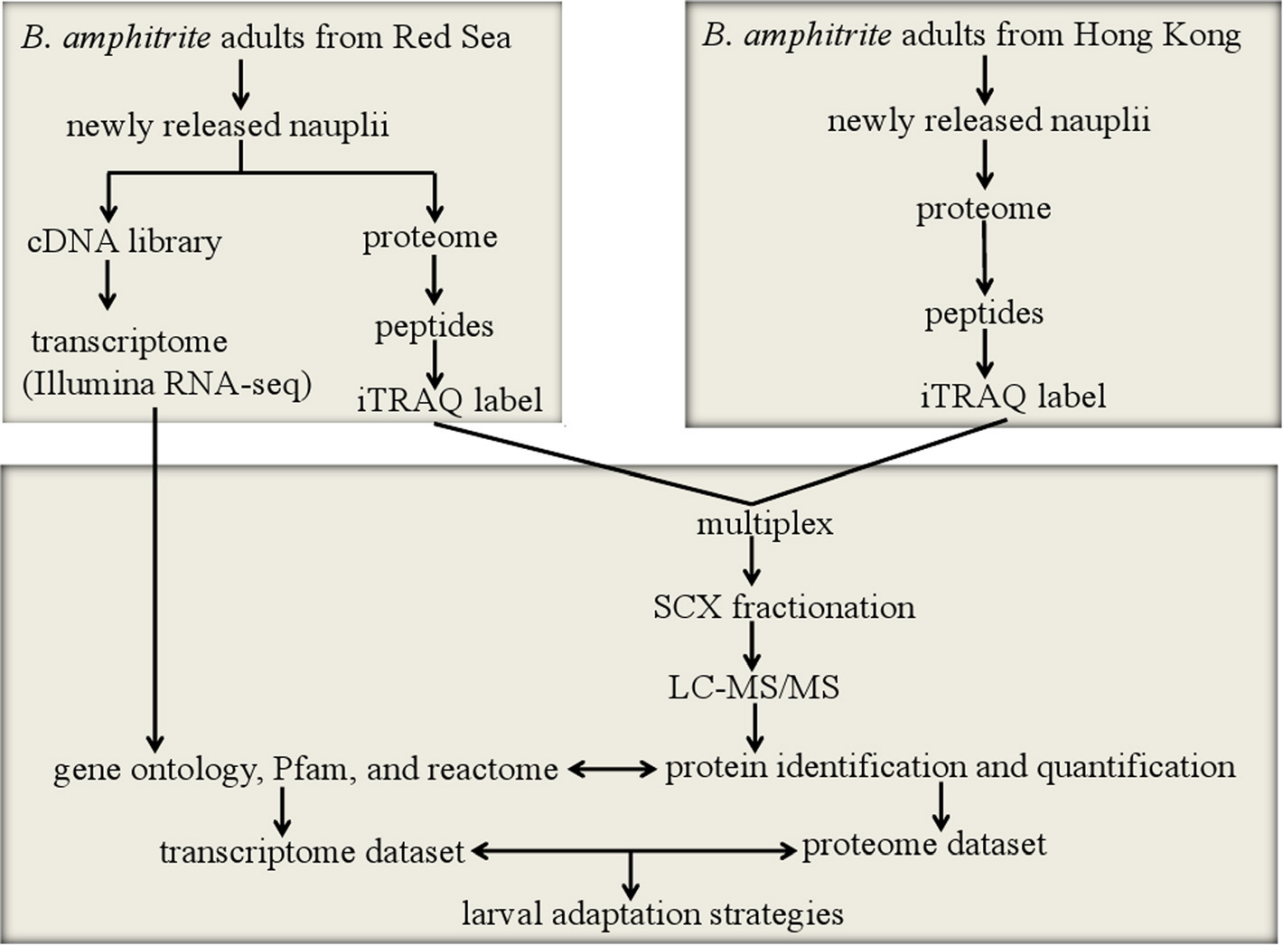
Schematic representation of the experimental design for the transcriptome and proteome analyses

**Fig. 2:**
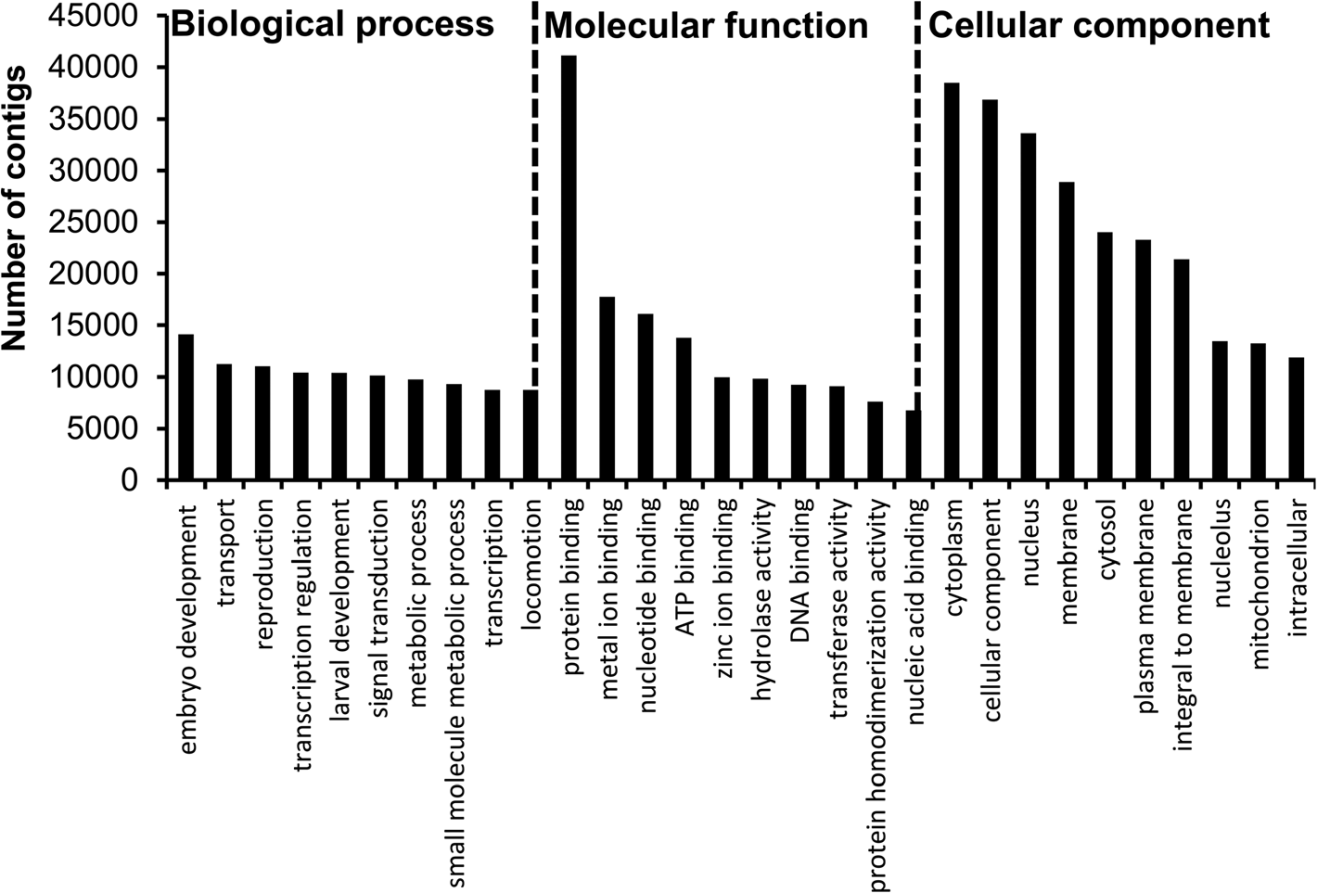
Gene ontology (GO) of assembled contigs of newly-released nauplii larvae of *B. amphitrite*. The top 10 categories for biological processes, molecular function and cellular components are shown

Figure 1 shows the experimental design used for the transcriptome and proteome analyses. Sequencing data statistics, assembly and annotation information for newly released nauplii of RS *B. amphitrite* are shown in Table 1. The transcriptome data were deposited in the NCBI BioProject ID: PRJNA256251 (http://www.ncbi.nlm.nih.gov/bioproject/256251). Approximately 170 million paired-end reads were obtained*.* Following trimming of low quality reads (Q-score < 20) and adapters, the number of reads was reduced to 151 million, with an average read length of 87 bp. Assembly of the reads using ABySS v1.3.4 and Trans-ABySS v1.4.4 yielded 479,922 contigs with an average contig length (N50) of 471 bp. Using GETORF we predicted 2,485,979 open reading frames (ORFs), of which 92,117 appeared in at least one blast hit. Blastp against a non-redundant (nr) database resulted in 89,151 contigs having at least one sequence homologous with other species, while no homolog was found for 390,771 contigs. An Interproscan v5.4 search resulted in 54,082 contigs having at least one annotated PFAM domain, while no domain was found for 425,840 contigs. In total, 76,263 contigs showed at least one gene ontology (GO) annotation while 403,659 contigs remained hypothetical, and 33,992 contigs showed at least one Reactome annotation, whereas no annotation was found for 445,930 contigs. A total of 1,613,892 were assigned to ‘biological process’ (BP), and 508,916 and 664,282 contigs were assigned to ‘molecular function’ (MP) and ‘cellular component’ (CC), respectively (Additional file 1: Table S1-S4). Figure 2 shows the top 10 categories of assembled contigs belonging to the BP, MF and CC categories. The GOs having the top 10 hits and contigs under the BP category were ‘embryo development’ (14091), ‘transport’ (11260), ‘reproduction’ (11047), ‘transcription regulation’ (10440), ‘larval development’ (10417), ‘signal transduction’ (10150), ‘metabolic process’ (9767), ‘small molecule metabolic process’ (9316), ‘transcription’ (8757) and ‘locomotion’ (8743).

**Table 1:** Sequencing data, assembly, and annotation statistics of RS newly released nauplii larvae of *B.amphitrite*

Raw read data	Before preprocessing	After preprocessing
Reads		
No. of reads	170,319,087	151,051,086
Average read length	101	87.17
Assembly		
No. of contigs	479,922	
Ave contig length (N50)	471	
Annotation		
Predicted open reading frames (ORFs)		2,485,979
ORFs with at least one blast result		92,117
Contigs with at least one homolog		89,151
Contigs with at least one annotated protein domain		54,082
Contigs with at least one GO annontation		76,263
Contigs with at least one REACTOME annontation		33,992

### Abundant transcripts, protein domains and Reactome pathways

Functional analysis of contigs revealed that transcripts related to translation, oxidative phosphorylation and cytoskeletal development were highly abundant. The top 40 abundant transcripts and the number of reads are shown in Additional file 2: Table S5. Elongation factor-1 alpha was ranked first (1,698,408 reads), followed by NADH dehydrogenase subunit 4 (1,100,166 reads). Notably, four transcripts encoding cytochrome oxidase subunit I were also well represented. Interestingly, 10 of the 40 most abundant transcripts encoded hypothetical or predicted proteins. Additional file 3: Table S6 lists the top 30 Pfam domains predicted in the transcriptome. We were able to map 26 pathways present in the Reactome database (Additional file 4: Table S7). These pathways were highly overlapping in terms of the GO BP (Fig. 2, Additional file 1: Table S2) and differentially-expressed protein categories (Table 2).

**Table 2:** Up-regulated proteins involved in stress response and osmoregulation in Red Sea newly released nauplii larvae of *B.amphitrite*

Accession no.	Contig no.^a)^	Protein description	Unique peptides	Sequence coverage (%)	Fold change^b)^ (RS/HK)	STDVE
		Stress response/defense/energy				
AAN74984.1	348386_5	70 kDa heat shock protein 70 kDa (HSP70)	3	28	2.07	0.078
XP_001951792.1	284615_1	heat shock 70 kDa protein 4 L ((HSP70-4 L)	2	31	2.78	0.043
XP_969171.1	221326_2	chaperonin (CPN)	1	34	1.71	0.283
AER95784.1	112034_2 (+1)	chaperonin containing TCP1subunit 4(CPN-TCP1-4)	2	30	1.6	0.07
BAG72108.1	333804_5	chaperonin eta subunit (CPN-eta)	3	29	1.6	0.028
XP_004206182.1	216349_6 (+2)	major egg antigen (MGN)	8	28	2.31	0.057
XP_001606473.2	235958_8	T-complex protein 1 subunit zeta- isoform 1 (TCP1)	2	20	2	0.043
BAA92814.1	379389_7	annexin IX-A (AnxIXA)	2	30	3	0.043
BAG41813.1	149743_6	cyclophilin A (CyPA)	4	44	2.21	0.088
XP_001521816.2	331314_1 (+2)	collagen alpha-3(IV) chain (Col4)	1	13	2.7	0.068
ABZ91537.1	11340_21	vitellogenin (VTG)	3	9	5.53	0.821
XP_002741413.1	9957_2	vitellogenin structural gene-1 (VTG1)	3	12	1.96	0.076
		Osmoregulation				
AEX07319.1	136693_6	Na+/K + −ATPase alpha subunit (H + K + αATPase)	6	21	1.85	0.168
AAA35578	356633_2	ATPase	2	50	2.03	0.017
XP_003817086.1	110200_6	sodium-dependent phosphate transport protein 2C(SLC2C)	3	60	4.35	0.2
XP_003244453.1	333233_4	v-type proton ATPase 116 kDa subunit a isoform 1(ATP6V0A1)	2	12	2.91	0.432
P31401.1	358029_1	v-type proton ATPase subunit B (ATPV0B)	2	64	3.05	0.367
ADI56517.1	263296_2	voltage-dependent anion channel 2 (VDAC2).	2	62	1.75	0.017
XP_004073305.1	259151_2	neuronal acetylcholine receptor subunit non-alpha-3 (α7-nAChR)	1	17	4.09	0.076
ADO27772.1	30169_8 (+1)	carbonic anhydrase (CA)	1	5	4	0.13
AFS60116.1	324487_3	selenoprotein M (SelM)	1	28	4.4	0.119
		Muscle contraction				
EFX86510.1	142581_8	myosin essential light chain (MLC)	1	16	2.65	0.249
XP_002425291.1	233725_2	paramyosin(PM)	1	9	2.71	0.057
XP_002432355.1	385389_2 (+1)	paramyosin, long form (PM-L)	1	32	1.9	0.139
EGI57423.1	295447_2	titin(TTN)	1	8	2.91	0.07
AAT77811.1	33648_2 (+1)	calpain B(CalpB)	1	35	1.84	0.035
		Development/morphogenesis				
ADV40137.1	350939_3	actin 5C (ACT5C)	2	60	3.6	0.07
XP_003696514.1	286277_1	actin, indirect flight muscle-like isoform 2(ACTIFM2)	2	96	2.29	0.135
XP_003696514.1	103842_3	actin, indirect flight muscle-like isoform 2 (ACTIFM2)	3	62	2.17	0.043
ACO11823.1	368856_3	actin, muscle (ACTM)	3	26	1.72	0.143
NP_001029376.1	358955_6	tubulin alpha-1C chain (TUBA1C)	2	15	3.01	0.204
XP_003220514.1	322186_3	tubulin alpha-1C chain (TUBA1C)	2	67	1.63	0.064
XP_001928233.1	258099_4 (+1)	tubulin alpha-1D chain (TUBA1D)	1	22	4.17	0
XP_624843.2	161549_1 (+1)	filamin-B (FLNB)	2	26	2.31	0.028
		Cellular metabolism				
ACX53990.1	291866_1 (+3)	ubiquinol-cytochrome c reductase complex (UQCCR)	1	13	1.83	0.057
EEZ99338.1	333062_1	cytochrome P450 6BQ13 (CY-P450 6BQ13)	1	8	4.13	0.224
XP_970215.1	359961_2	cytochrome P450 CYP6BK17 (CY-P450 6BQ13)	1	2	3.15	0.028
XP_004079962.1	302118_1 (+1)	dehydrogenase/reductase SDR family member 4(DHRS4)	2	44	3.21	0.132
ZP_09403919.1	328617_1	short chain oxidoreductase (SCOR)	2	25	2.14	0.043
XP_003204363.1	272396_1	reticulon-4-interacting protein 1, mitochondrial (RTN4IP1)	1	26	3.64	0.081
ZP_01905843.1	388126_2	oxidoreductase, short chain dehydrogenase/reductase family protein(SDR)	1	13	2.38	0.078
XP_003701373.1	142763_2	trans-1,2-dihydrobenzene-1,2-diol dehydrogenase(DHDH)	1	7	1.83	0.028
		Signaling pathways				
XP_966512.1	40595_3	GTP-binding nuclear protein Ran isoform 1 (RanGTPBP1)	6	37	1.93	0.067
XP_002432661.1	92938_6	GTP-binding protein SAR1B (GTP-BP-SAR1b)	1	75	4.8	0.247
XP_001945117.1	198588_2	guanine nucleotide-binding protein G(i) subunit alpha(GNBPα)	1	6	1.93	0.067
NP_001040272.1	194501_4	receptor for activated protein kinase C isoform 2 (RACK2)	3	41	1.63	0.092
NP_001037203.1	238662_1 (+1)	innexin 2 (INX2)	3	23	1.96	0.073
XP_003488474.1	362217_1	epidermal growth factor receptor substrate 15 (EGFR15)	1	19	2	0.159

^a)^ Refer to contig no. in transcriptome database

^b)^ Averaged expression values derived from independent replicates of Red Sea (RS)/Hong Kong (HK) samples

**Fig. 3:**
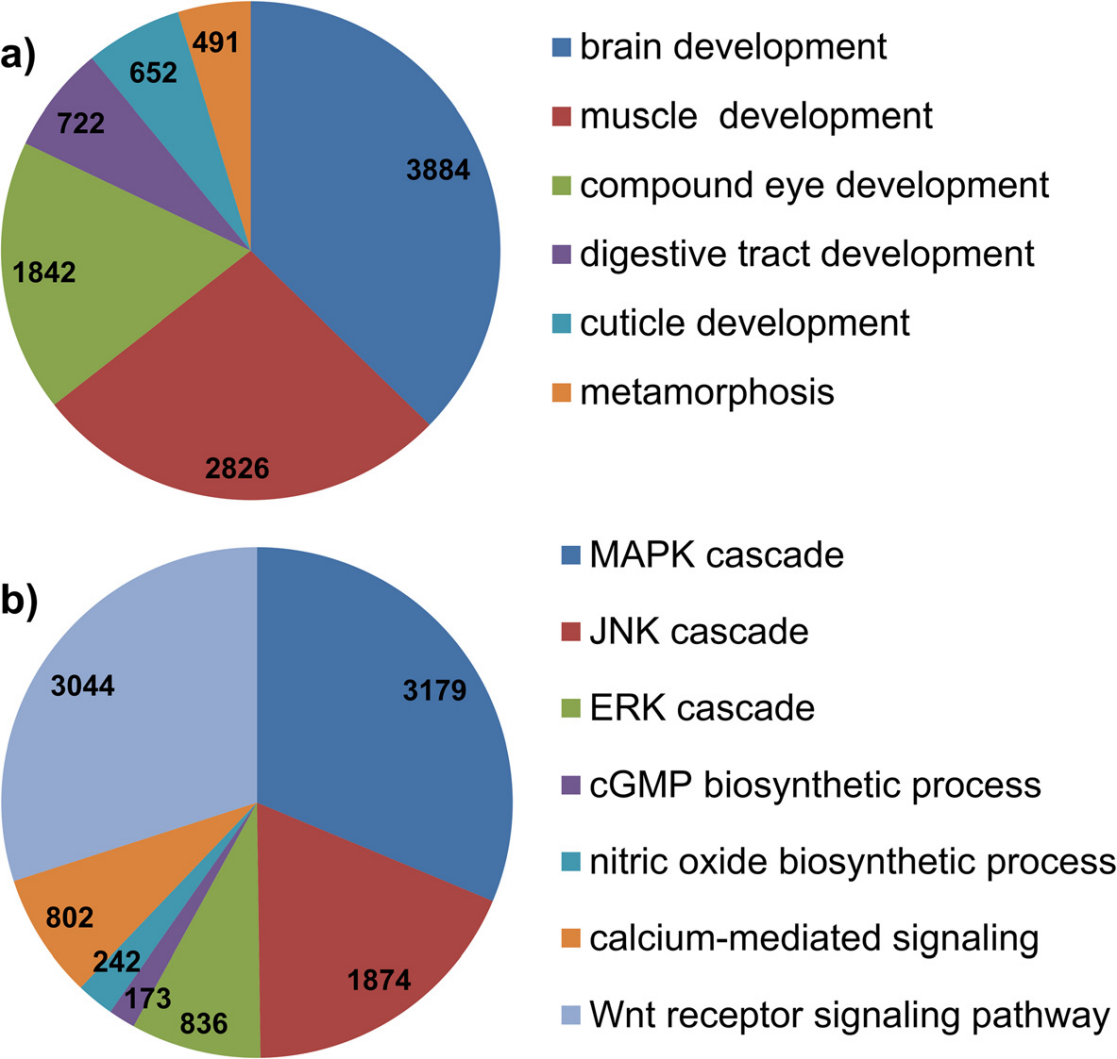
Selected genes involved in (**a**) larval development and (**b**) signaling mechanisms. The contig numbers inside the pie chart were obtained from the GO biological functions listed in Supplementary Table S1

**Fig. 4:**
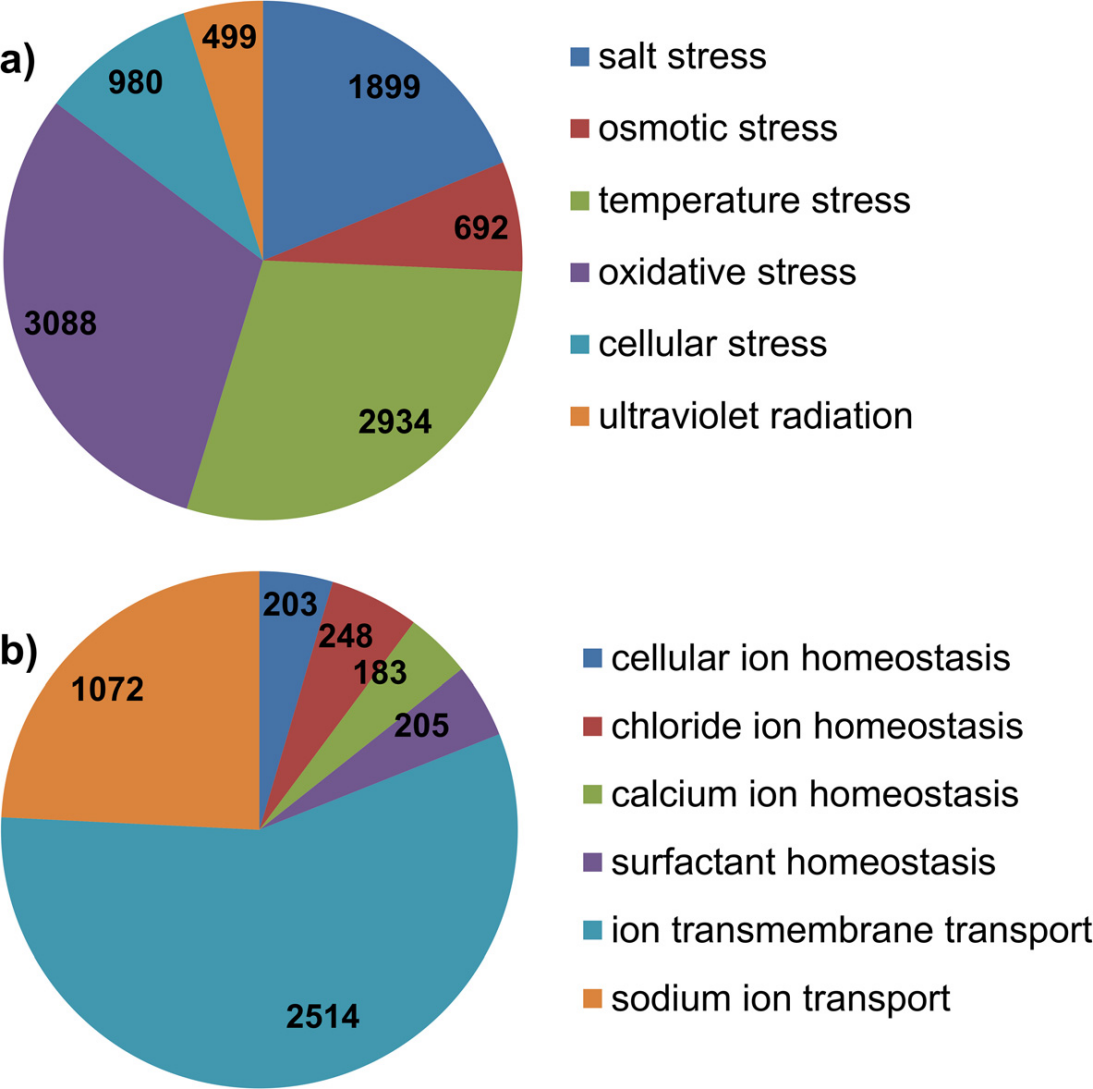
Selected genes involved in (**a**) response to environment stressors and (**b**) osmoregulation. The contig numbers inside the pie chart were obtained from the GO biological functions listed in Additional file 11: Figure S1

A large number of genes related to larval development were found to be expressed (Fig. 3a), among which those for brain (GO:0007420; 3884 contigs), muscle (GO:0007517; 2826 contigs), eye (GO:0048749; 1842 contigs), digestive track (GO:0048565; 722 contigs) and cuticle (GO:0040003; 652 contigs) were dominant. Notably, 491 genes related to metamorphosis (GO:0007552) were also identified. A substantial number of genes assigned to signaling pathways involved in larvae development were also detected (Fig. 3b). We found that a large number of genes involved in stress and osmoregulation were over-represented. Genes expressed in response to high salinity, high temperature, cellular stress and ultraviolet (UV) light exposure were identified (Fig. 4a). We also identified several genes involved in homeostasis (Fig. 4b).

### The larvae proteome

A total of 1264 proteins from HK and RS nauplii were identified based on 4434 unique peptides and 5286 unique spectra (Additional file 5: Table S8). Proteins were quantified using HK larvae as a reference (reporter ion intensities were adjusted to 1; fold change > 1.5). Among these proteins, 170 showed differential expression in RS larvae, based on an average of 1.9 unique peptides, 2.22 spectra and 22.76 % sequence coverage. Of these proteins, 89 were up-regulated and 81 were down-regulated in RS larvae (Tables 2 and 3). Additional file 6: Table S9, Additional file 7: Table S10 and Additional file 8: Table S11 shows the number of peptides and the spectra, sequence coverage, peptide sequences and fold changes for each differential protein.

**Table 3:** Down-regulated proteins involved in homeostasis and energy metabolism in Red Sea newly released nauplii larvae of *B.amphitrite*

Accession no.	Contig no. ^a)^	Protein description	Unique peptides	Sequence coverage (%)	Fold change^b)^	STDVE
		Detoxification				
AGA83299.1	143917_1	glutamine synthetase (GS)	1	6	2.1	0.046
EKC40774.1	381680_1	glutaredoxin(GRX)	1	15	2.34	0.021
EFX81633.1	352718_6	glutathione S-transferase (GST)	2	18	2.24	0.053
NP_001108460.1	176458_3	glutathione S-transferase epsilon 4 (GST4)	4	44	2.24	0.014
XP_537038.2	68758_2	glutathione S-transferase Mu 1 isoform 1 (GST Mu1)	2	9	1.73	0.098
XP_003693286.1	382037_1	aldehyde oxidase 2 (AO2)	1	6	2.01	0.129
EHJ73729.1	382498_5	alcohol dehydrogenase (ADH)	1	4	1.77	0.028
AEE36486.1	326926_1 (+1)	protein disulfide isomerase 2 (PDI2)	6	47	1.6	0.017
AFL70280.1	281344_8 (+1)	protein disulfide-isomerase A6 (PDIA6)	1	8	1.83	0.09
		Transport				
XP_002431951.1	15248_12	dynein heavy chain, cytosolic (DHC)	1	1	5.16	0.228
XP_002426223.1	129454_5	mitochondrial import inner membrane translocase subunit Tim23 (TIMM23)	1	9	1.75	0.043
EFX90424.1	283903_1	mitochondrial inner membrane protein (MIM)	2	14	1.84	0.128
XP_967948.2	299347_2	bumetanide sensitive NaK2Cl cotransporter isoform 1 (NKCC2)	1	9	1.75	0.095
ADM64456.1	378620_1 (+1)	fatty acid binding protein (FABP)	5	44	1.93	0.028
XP_393519.1	237152_4 (+3)	fatty acid-binding protein, adipocyte (FABPA)	1	11	1.92	0.177
XP_001601236.1	34337_3 (+1)	nuclear transport factor 2 (NTF2)	1	9	2.06	0.017
XP_973673.1	301328_8	vesicle associated protein (VAP)	3	5	1.78	0.067
XP_002737711.1	360183_6 (+2)	coated vesicle membrane protein- isoform 2 (VAMP-2)	1	11	1.91	0.014
EGF79988.1	198964_4	peptidyl-prolyl cis-trans isomerase (PPI)	2	94	2.81	0.123
ACJ05313.1	307444_1 (+1)	leucine aminopeptidase(LAPs)	1	9	1.74	0.064
		Glycolysis/gluconeogenesis				
ACD75898.1	360305_3	enolase (EN)	1	43	2.21	0.159
AAS02310.1	286350_2 (+1)	glyceraldehyde 3-phosphate dehydrogenase (GPDH)	1	77	5.75	0.043
AGC97435.1	281394_3	glycogen phosphorylase (GPs)	1	12	1.66	0.057
XP_002400796.1	278577_6	GDP-mannose pyrophosphorylase (GDP-MP)	1	3	2.1	0.043
XP_003393705.1	130725_2 (+2)	transketolase 2 isoform 1 (TKT2)	2	14	1.7	0.014
Q91XV4.1	312847_2	L-xylulose reductase (XRs)	1	11	1.71	0.028
BAJ23881.1	360259_3	fructose 1,6-bisphosphatase (FDPase)	2	21	3.54	0.035
ACH81781.1	242946_2	fructose 1,6-bisphosphate aldolase (FBPA)	7	32	2.05	0.014
XP_001601687.1	383275_9	phosphoglucomutase-2- isoform 1 (PGM 2)	1	4	1.66	0.057
XP_003240970.1	383186_3	pyruvate carboxylase, mitochondrial- isoform 3(PCm3)	2	15	1.91	0.095
EKC25154.1	284278_2 (+1)	UTP--glucose-1-phosphate uridylyltransferase (GalU)	1	7	2.28	0.035
XP_001847482.1	255952_3	UTP-glucose-1-phosphate uridylyltransferase 2 (GalU2)	2	11	2.53	0.176
		Actin-binding proteins				
XP_001944078.1	370072_2	vinculin (VCL)	1	19	1.62	0.035
CCI71880.1	97015_4	gelsolin (GSN)	1	15	1.55	0.039
XP_001657431.1	377569_1 (+1)	gelsolin precursor(GSNP)	2	18	1.71	0.043
XP_003691411.1	216_3	plastin-3(PLS3)	2	10	1.71	0
JAA59814.1	237239_4	villin-1 (VIL1)	3	27	1.81	0.028

^a)^ Refer to contig no. in transcriptome database

^b)^ Averaged expression values derived from independent replicates of Red Sea (RS) /Hong Kong (HK) samples

### Differences in proteome expression between RS and HK larvae

**Fig. 5:**
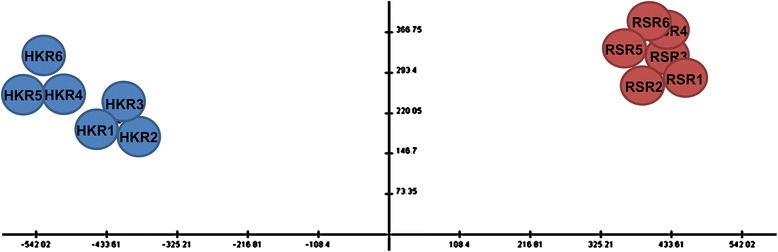
2-D view of the principal component analysis of 1264 identified proteins in two natural larval populations. Log2-transformed fold change ratios from six replicates were used for the analysis. HKR: replicates of Hong Kong larvae; RSR: replicates of RS larvae

**Fig. 6:**
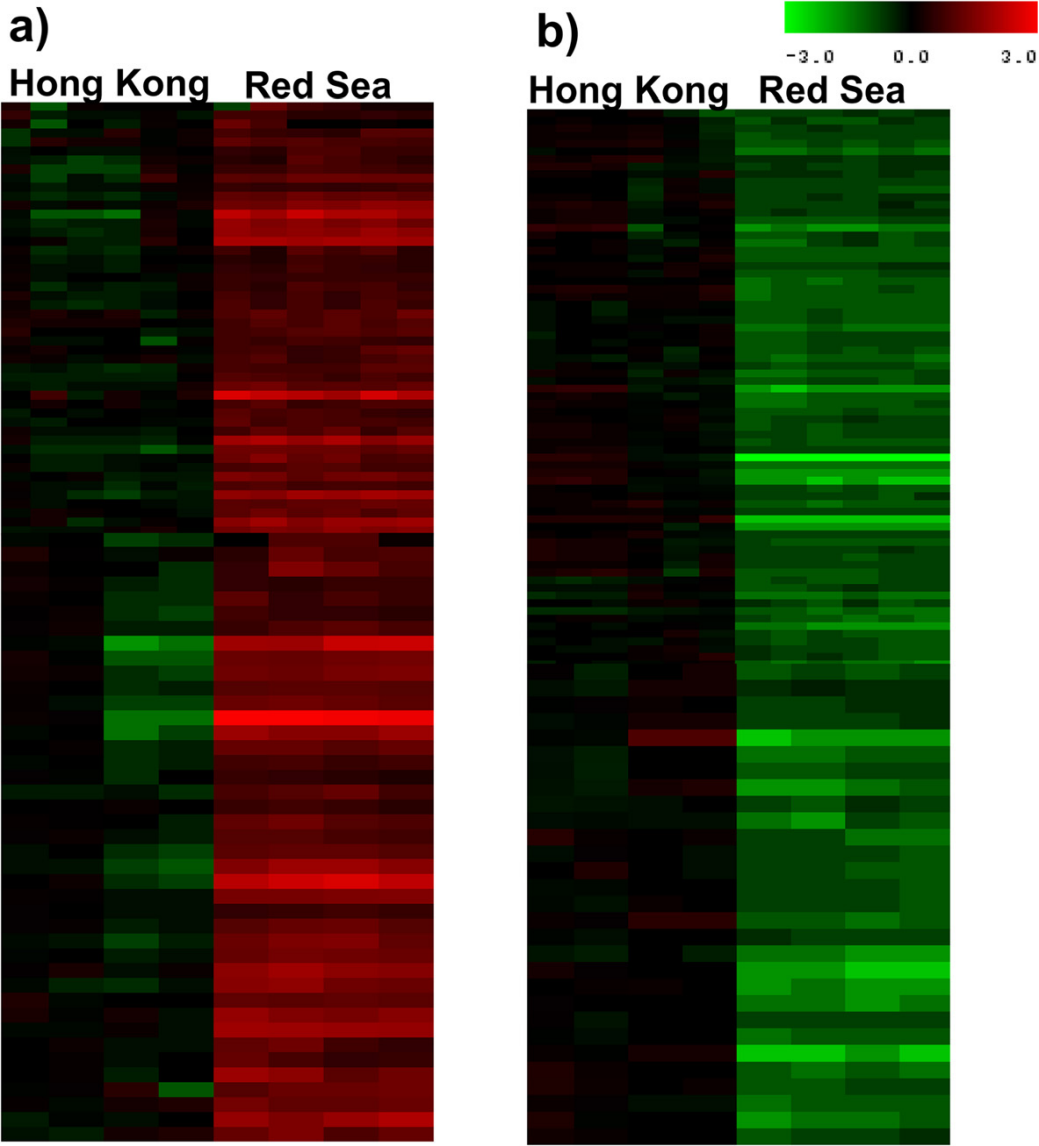
Hierarchical clustering of differentially-expressed proteins in Hong Kong and Red Sea larvae of *B. amphitrite*. **a**) Hierarchical cluster 1, **b**) Hierarchical cluster 2. Red: up-regulation; green: down-regulation. The horizontal axis indicates the replicates in the order R1 to R6

**Fig. 7:**
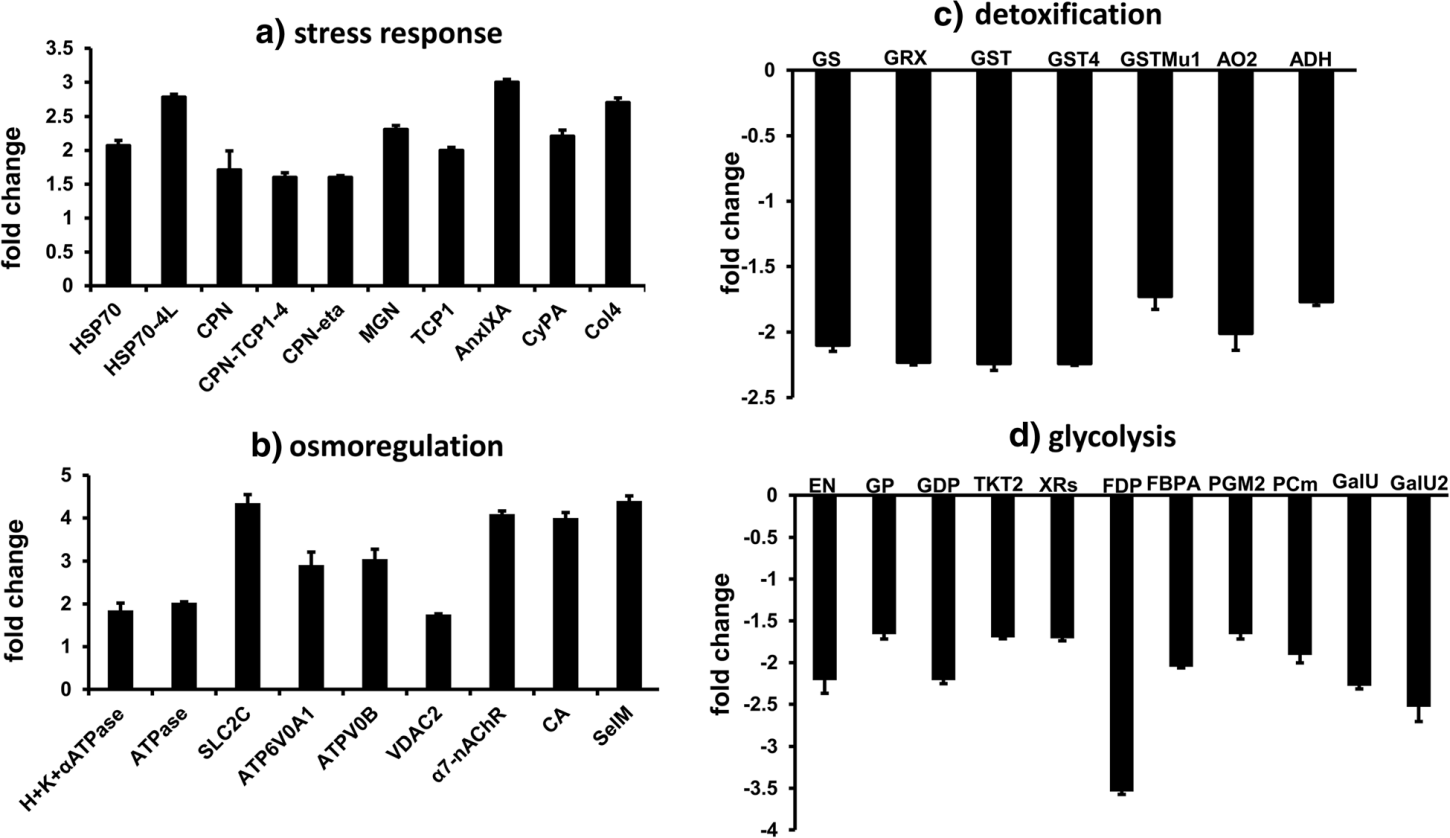
Proteins involved in the stress response (**a**) and osmoregulation (**b**) were up-regulated in Red Sea *B. amphitrite* larvae, whereas detoxification (**c**) and glycolysis (**d**) proteins were down-regulated. The full protein names are shown in Tables 2 and 3

Principle component analysis (PCA) of the proteome datasets showed clear separation of the HK and RS samples (Fig. 5), and hierarchical clustering analysis revealed two clusters of down-regulated (green) and up-regulated (red) proteins in the RS larvae that were distinct from those in the HK larvae (Fig. 6). A slight variation in expression pattern between the replicates (R1–R3 and R4–R6) of HK larvae was observed. It is important to note that the adult barnacles were collected from wild natural conditions. Habitat conditions could differ substantially because of factors including origin, age, gender and physiological state. It is difficult to account for uncontrolled sources of variation among specimens collected from natural habitats at the same time in the same area. However, such variability may not have a significant impact as the average individual protein expression was only slightly different among replicates. Furthermore, the average coefficient of variation (CV) among replicate samples from HK was lower (within the standard range for iTRAQ experiments), and not enough to mask the overall difference in the expression patterns of HK samples. Table 2 and Additional file 6: Table S9 lists the up-regulated proteins and their fold change values. Nine stress-activated proteins were up-regulated in the RS larvae relative to the HK larvae (Fig. 7a). The expression of osmoregulatory proteins was also significantly increased in the RS larvae (Fig. 7b). Additional file 7: Table S10 lists some of the down-regulated proteins and their fold change values. In RS larvae 9 proteins involved in cellular detoxification (Fig. 7c) and 11 involved in glycolysis and gluconeogenesis were down-regulated (Fig. 7d). Notably, all 5 actin-binding proteins, including gelsolins, vinculin, plastin-3 and villin-1 were also down-regulated.

### Correlation between the proteome and transcriptome of RS larvae

The number of genes in RS larvae of *B. amphitrite* that were identified in each functional category, and their corresponding protein expression patterns, are shown in Table 4 and Additional file 9: Table S12. The profiles of the two datasets revealed overlap in the majority of abundant transcripts and differentially expressed proteins. These categories, which included stress response, and development and signaling pathways, showed similar trends of expression at both the transcript and protein levels. We identified 30 stress response proteins from the proteome analysis and 16,367 contigs in the transcriptome; these belonged to various categories of stress. Similarly, the ‘developmental’ category showed 34 proteins and 24,597 transcripts, while ‘signaling pathways’ showed 18 proteins and 18,171 transcripts in the respective datasets. Further, the proteins of binding and catalytic activity were also equally represented in the proteome and the transcriptome. Overall, abundant proteins showed high degree of correlation with the corresponding transcripts.

**Table 4:** Parallel comparison of protein regulation to their corresponding transcript abundance in Red Sea larvae of *B.amphitrite*

Protein category	No. of proteins identified	Protein regulation	Transcripts abundance(no. of contigs)
Stress	12	up-regulation	10092
Osmoregulation	9	up-regulation	4425
Detoxification	9	down-regulation	1850
Cytoskeleton	8	up-regulation	11615
Actin binding	5	down-regulation	3916
Muscle development	5	up-regulation	323
Cuticle development	10	n/a	735
Locomotion	6	dual regulation	8743
Apoptosis	12	dual regulation	8561
Signaling pathways	6	up-regulation	10150

## Discussion

### Deep sequencing of the larval transcriptome

The transcriptome analysis yielded approximately 150 million reads and 479,922 contigs (N50*,* 471 bp) for the RS larvae, including 92,117 unique predicted ORFs, and constitutes the largest available sequence dataset for nauplii larvae. Chen et al. (2011) obtained 630,845 reads using 454 pyrosequencing and 23,451 contigs from pooled larvae and adult samples of *B. amphitrite* from HK seawater [13], while De Gregoris et al. (2011) generated 575,666 reads from nauplius, cyprid and adult stages of *B. amphitrite* [15]. More recently, Lin et al. (2014) obtained 77,528,326 reads and 104,610 contigs from the prosoma, and 59,244,468 reads and 123,534 contigs from the base of the barnacle *Tetraclita japonica formosana* [16]. The sequencing depth of the present study provides much greater read coverage than the previous studies. Using this in-house transcriptome database we successfully used mass spectrometry to identify 1264 proteins; of these, 170 showed distinct expression patterns between the HK and RS larval populations. We also identified a large number of transcripts related to development, locomotion and signal transduction.

### Molecular basis of larval development

The abundant expression of cuticular proteins in both the transcriptome and proteome datasets may reflect the importance of exoskeleton dynamics during development. The barnacle body is covered with exoskeleton, which is composed of carbohydrates and proteins [28]. During molting the larvae partially resorb the cuticle to provide space for newly forming tissues. The larval proteome showed that several structural proteins were differentially expressed. For example, myosins, titin, actin, tubulins, talin-1 and fasciclin-1 were up-regulated in RS larvae (Table 2), whereas settlement pheromone, cement secretion protein 2, retinol dehydrogenase 13 and NMDA receptor protein 1 were down-regulated (Table 3). This is consistent with our previous reports showing differential expression of cytoskeletal proteins during development in polychaetes [29–32]. The myosins and titins contribute to muscle development and the contraction of striated muscle. In barnacles, striated muscles in antennules and thoracopods help in locomotion and sensory detection for substratum attachment [33]. Barnacle cyprid-specific protein 2 (BCS-2) is abundantly expressed in cyprids, but decreases following larval attachment [34]. The protein BCL-4 is less abundant in young cyprids, but gradually increases in older cyprids. Apoptosis and protein degradation occur spontaneously during larval development and metamorphosis [35], and are critical in the removal and subsequent degradation of early larval structures, and in cellular and tissue remodeling. In the present study we found several differentially expressed proteins related to apoptosis. Our previous proteomic analyses identified several apoptosis proteins in early stages of larval development in barnacles [19].

### Signal transduction pathways

Genes that participate in the MAPK cascade, cGMP, nitric oxide (NO) biosynthesis, calcium and Wnt pathways were identified in the present study. Our previous studies showed that in barnacles these pathways are closely associated with larval settlement and metamorphosis. For example, p38-mediated MAPK signaling has been found to be crucial for larval attachment in barnacles [36, 37]. Zhang et al. (2012) found that NO regulates attachment of barnacle larvae by modulating cGMP signaling [38]. It appears that NO signaling contributes to stress-activated responses (in the present case, up-regulation of heat shock proteins), and thereby influences larval survival under unfavorable environmental conditions. Based on our findings, we hypothesize that MAPK, NO, cGMP and calcium signaling are important for larval development and stress tolerance in larvae of *B. amphitrite*. Furthermore, the proteome data showed up-regulation of six proteins related to G protein signaling or protein kinase. Among these, GTP-binding nuclear protein (RanGTPBP1), GTP-binding protein SAR1B (GTP-BP-SAR1b) and guanine nucleotide-binding protein (GNBPα) belong to G protein signaling, whereas receptor for protein kinase C (RACK2), innexin 2 (INX2) and epidermal growth factor receptor (EGFR15) belong to the protein kinase and calcium receptor-mediated signal transduction cascades. Chen et al. (2014) reported up-regulation of the G protein-coupled receptor in barnacle cyprid larvae [20], and G protein receptors bind to the metamorphic cue, while the protein kinase independent pathway participates in settlement signaling.

### Adaptive strategies under extreme marine conditions

Adult barnacles live in various habitats and are subject to differing selection pressures that could have a major influence on the survival strategies of their offspring (in this case nauplii) [39, 40]. Temperature and salinity influence larval recruitment and the survival of marine invertebrates [41, 42]. In addition, the intertidal zone is highly variable with respect to temperature conditions and among different marine environments [43]. For example, in summer the offshore RS and HK water temperatures average 30.8 °C and 28 °C, respectively [27, 44]. Nauplii may have developed remarkable adaptive mechanisms for survival in the pelagic stage and display a variety of survival strategies. The higher expression levels among stress response genes in RS larvae, and the up-regulation of stress-activated proteins including heat shock proteins (HSPs), chaperonins (CPNs), major egg antigen (MGN), T-complex protein (TCP1), annexin (AnxIXA), cyclophilin (CyPA) and collagen alpha (Col4) suggest the development of adaptive strategies to cope with extreme conditions. This observation is consistent with previous studies showing significant up-regulation of HSPs in barnacle larvae challenged with antifoulants [16, 23]. The cellular response of larvae to stress leads to abundant expression of genes encoding HSPs. Some HSPs act as molecular chaperons (CPNs, MGN, TCP1) by modulating the correct folding of other proteins under stress, and are involved in various developmental signaling pathways, and assembly and transport mechanisms [45, 46]. For instance, in ascidians and gastropods, HSP90 activity regulates the repression of larval metamorphosis by nitric oxide signaling [47]. AnxIXA expression patterns have been shown to change significantly when cells undergo proliferation or differentiation [20]. The differential expression of AnxIXA in RS nauplii and juveniles of *B. amphitrite* could contribute to anti-inflammatory responses. CyPA secreted by cells in response to inflammatory stimuli is known to play an important role in protein folding. We identified a large number of genes involved in osmotic stress tolerance. Furthermore, the RS larval proteome displayed dramatic up-regulation of osmoregulatory proteins including H + K + αATPase, v-type proton ATPase, phosphate transport protein (SLC2C), voltage-dependent anion channel (VDAC2), neuronal acetylcholine receptor (α7-nAChR), carbonic anhydrase (CA) and selenoprotein (SelM) (Table 2). During barnacle larval development, biomineralization and osmoregulation facilitate larval tolerance to a wide range of osmotic stresses via active ion transport [48]. CA is widely distributed in barnacle tissues, and is involved in the synthesis of calcium carbonate [20] and the molting cycle in crustaceans. In other marine species osmotic stress affects the expression patterns of the proteins ATP6V0A1 and ATPV0B, and Na + −K + −ATPase and V-H + −ATPase are required for ion regulation [49]. The sodium-phosphate symporter SLC2C plays a role in osmoregulation by absorbing phosphate from interstitial fluid. The Ca^2+^ transport regulator VDAC plays an important role in regulating the exchange of ions and solute molecules in mitochondria, and α7-nAChR facilitates the opening of ion channels across the plasma membrane. Consequently, we believe that under extreme environmental conditions larvae may be protected by abundant expression of osmoregulatory proteins that maintain cellular homeostasis, and that the large number of genes involved in various homeostasis mechanisms (including for chloride, calcium and sodium ions, and regulation of ion transmembrane and surfactant concentrations) are probably involved in osmoregulation (Fig. 4b).

### Metabolic depression

Under extreme environmental conditions marine invertebrates typically show greater metabolic depression by minimizing energy loss [50, 51]. It has been estimated that protein translation alone may consume up to 50 % of cellular energy; by suppressing metabolism, energy can be diverted to synthesis of stress tolerance proteins [52]. Metabolic depression may lead to functional cellular modification, post-translational modification of proteins (phosphorylation), and maintenance of homeostasis by ion transport. Consistent with these observations*,* in RS larvae we found down-regulation of 81 proteins involved in various metabolic processes (Table 3). For instance, glutathione S-transferases (GSTs) are antioxidant isoenzymes that provide cellular defense against oxidative stress and harmful chemicals [53]. Han et al. (2013) reported increased expression of GSTs in barnacle cyprid larvae following exposure to the antifoulant meleagrin [23], suggesting a role in chemical detoxification. GST also regulates apoptosis by inhibiting kinases, thereby affecting downstream signaling pathways. It has been shown in barnacle cyprid larvae that proteins involved in glycolysis and gluconeogenesis are differentially expressed, suggesting roles in maintaining homeostasis [20, 23]. Fatty acid binding proteins (FABPs) and fructose 1,6-bisphosphatase (FDPase), which are involved in the synthesis of byproducts of glycolysis and gluconeogenesis, have been identified in barnacle larvae [20]. Membrane proteins play an important role in the transport of solutes and toxic materials across the cell membrane. In particular, mitochondrial inner membrane proteins (MIM and TMIM 23) transport metabolites across the membrane in a highly controlled manner [54]. FABPs act as carrier proteins for fatty acids and other lipophilic substances [55]. The regulation of energetically expensive transport systems appears to be an important strategy used by RS larvae to survive in extreme conditions. The actin binding proteins may regulate cytoskeletal dynamics in developing larvae. It is hypothesized that larval tolerance of stress is regulated by complex stress response mechanism and strategies, and metabolic depression is probably more diverse and complicated than previously assumed. We believe this study is of international significance in advancing marine biology, as most previous studies have focused on response to specific environmental factors in controlled environment; it also highlights the importance of studying the cumulative effects of environmental conditions in natural habitats.

## Conclusion

The transcriptome and proteome data generated in this study provide a valuable resource that will enhance molecular studies of crustacean larvae, which are of ecological and economic importance. The large differences found in the protein expression patterns of larval populations from the two contrasting environments were striking. The genes, proteins and signaling pathways identified may play crucial roles in the development of nauplii in the RS environment. For example, the abundant expression of cuticular and muscle proteins in nauplii is critical in the molting cycle, and in muscle development that influences swimming and sensory detection. We also identified genes that participate in several biochemical pathways that are important for larval settlement, metamorphosis and the stress response in barnacles. Specifically, heat shock proteins, chaperonin, major egg antigen, annexin and NO signaling participate in stress-activated responses, and thereby contribute to larval strategies for survival in the RS environment. Proteins involved in osmoregulation, including ATPase, transport proteins, acetylcholine receptor and carbonic anhydrase facilitate larval tolerance to osmotic stress. Overall, the findings of this study contribute significantly to knowledge of larval development and adaptation in extreme conditions, and the settlement biology of crustaceans.

## Methods

### Environmental conditions

Marine environmental data were collected from each sampling location. Measurements were carried out using a YSI Professional Pro Plus Multiparameter meter (YSI, Ohio, USA), which was calibrated prior to use. The probe was immersed in the water at the designated locations, and measurements were recorded after 2–3 min; it was rinsed with fresh water between measurements and locations. The average values of salinity, temperature, pH and dissolved oxygen were 42.31 psu, 34.23 °C, 8.2 and 5.06 mg/L, respectively. The values for HK water for salinity, temperature, pH and dissolved oxygen were 31.3 psu, 29.16 °C, 8.13 and 6.53 mg/L, respectively (Additional file 10: Table S13).

### Barnacle collection

Barnacles were collected from the intertidal zone between August 2012 and November 2012. Adult *B. amphitrite* barnacles attached to hard rocks were collected from the intertidal rocky shore of the King Abdullah University of Science and Technology, Thuwal, Kingdom of Saudi Arabia (27°18′168″N, 35°05′661″E). Approximately 100 collected adults were washed to remove attached debris and sediment, and were kept in a 10 L tank containing aged filtered sea water. The tank was covered with black plastic sheet, but had a small opening enabling light to penetrate, as a means of attracting newly released larvae. Newly hatched nauplii (hereafter, newly released RS larvae) were collected at 30-min intervals using a pipette until sufficient larvae were obtained for subsequent studies. Excess water was removed and approximately 3–4 × 10^3^ larvae derived from independent replicates were separated into two groups, one of which was used for transcriptome analysis and the other for proteome analysis (Additional file 11: Figure S1).

Approximately 100 individuals of adult *B. amphitrite* barnacles were collected from concrete columns at Pak Sha Wan, HK (22°21′45″N, 114°15′35″E), and placed in a 10 L tank containing filtered seawater (FSW; 0.22 μm) to induce the release of larvae. Approximately 3–4 × 10^3^ newly hatched nauplii (hereafter, newly released HK larvae) were collected as described above, and transferred overnight on dry ice to the King Abdullah University of Science and Technology. No significant size difference in the adults from the two populations was observed. On arrival the larvae were thawed, washed in seawater, and used for proteome analysis.

For the RS larval samples the total RNA was extracted from 600 to 700 larvae using TRIzol reagent (Invitrogen)*,* following the manufacturer's instructions. TURBO DNA-free treatment was performed using RNeasy spin columns (Qiagen). The RNA was purified and eluted in 14 μl of MilliQ water. The purity was checked using a NanoDrop ND-1000 UV–vis spectrophotometer (NanoDrop Technologies). The integrity of the extracted RNA was then checked using an Agilent 2100 Bioanalyzer (Agilent Technologies), and confirmed on a 1 % agarose gel.

### Library preparation and Illumina sequencing

cDNA library preparation was performed according to the Illumina TruSeq standard RNA Seq library preparation protocol, and sequencing was performed using an Illumina HiSeq 2000 genome sequencer. Briefly, the quantity of total RNA was measured using a Qubit*®* 2.0 fluorometer (Invitrogen). rRNA was removed using the Ribo-Zero Magnetic Gold Kit (Epicenter, Illumina). The RNA was subjected to thermal fragmentation*,* and first strand cDNA was synthesized using Super-Script III reverse transcriptase (Invitrogen). The cDNA was converted into double stranded DNA using the Second Strand Making Master Mix (Illumina). After dA-tailing, ligation of adaptors and purification, library amplification (15 cycles) was performed using a thermal cycler (Applied Biosystems). The libraries were purified using AMPure XP beads (Beckman Coulter, Inc.). Library dilution and pooling was carried out as described in the Illumina standard protocol. A quantity of 7.5 picomoles of DNA was used for cluster generation (CBOT Illumina) and sequencing.

### Assembly and annotation

Low quality reads (Q-score < 20) and adapters were trimmed using java scripts designed in-house. Reads were assembled using ABySS v1.3.4 [56] with setting parameters of every odd *k*-mer from 45 to 85, and consolidated using Trans-ABySS v1.4.4 [57]. Contig redundancy was reduced using CD-HIT-EST with the threshold sequence identity set to 0.99, to discard contigs with < 10 bp mismatches per 1 kb [58]. To obtain coding amino acid sequences we used GETORF to translate contigs from start to stop codons for all six possible reading frames [59]. Two approaches were used for functional annotation of contigs. First, homologous proteins from other species were searched using blastp against a non-redundant (nr) database (minimum bit score threshold 50; match length 20 amino acids). Second, the PFAM domain of the proteins was searched using Interproscan v5.4 [60]. Gene ontology (GO) and Reactome pathway information from homologous genes was transferred to barnacle contigs using a transcript designed in-house.

### Larval proteome extraction, digestion and iTRAQ labeling

The HK and RS larvae were suspended in 8 M urea and protease inhibitor (Roche Diagnostics, Germany) and homogenized (Wheaton homogenizer, USA). The homogenate was sonicated at 4 W using five × 1 s bursts (Q Sonica, LLC, USA), and the protein concentration was measured using a 2-D Quant kit (GE Healthcare, United Kingdom). Protein (100 μg) from each sample was reduced, alkylated, and diluted by a factor of 8 using 50 mM iTRAQ dissolution buffer as previously described [61]. The protein was digested using a trypsin–protein ratio of 1:50 for 14–16 h at 37 °C (Promega, USA). The digest was desalted using Sep*-*Pak C18 Vac cartridges (Water Corporation, USA), and labeled using the iTRAQ Reagents Multiplex (4plex) Kit (Applied Biosystems, USA), according to the manufacturer’s protocol. Briefly, dried peptides were reconstituted in 30 μL of resolubilization buffer, and 70 μL of ethanol was added to the iTRAQ reagents; the reagents 114 and 115 were labeled to HK larval peptides, and reagents 116 and 117 were labeled to the RS larval peptides. Labeling was carried out at room temperature for 60 min, and the labeled peptides were multiplexed and dried prior to fractionation.

### Peptide fractionation coupled with mass spectrometry

The iTRAQ-labeled peptides were suspended in 85 μL strong cation exchange chromatography (SCX) buffer A, and centrifuged at 10,000 rpm for 5 min at room temperature. The supernatant containing peptides was transferred to liquid chromatography (LC) sample vials and fractionated using an Accela 1250 LC system (Thermo Scientific, USA). The SCX-fractionated peptides were desalted using Sep*-*Pak C18 Vac cartridges and resuspended in 20 μL of LC sample buffer (97 % H_2_0, 3 % ACN, 0.1 % formic acid), centrifuged at 10,000 rpm for 5 min, and then transferred to LC-MS sample vials. A total of 15 SCX fractions were run in a LTQ-Orbitrap Velos mass spectrometer (Thermo Scientific*,* Germany) coupled to a Proxean Easy-nLC liquid chromatography system (Bruker, Denmark), as described previously [61].

### Protein identification

Raw MS data were processed following a previously described procedure [62]. Briefly, higher-energy collisional dissociation (HCD) and collision-induced dissociation (CID) spectra were extracted independently using Proteome Discoverer 1.2 software (Thermo Scientific), and processed using a script designed in-house. Mascot generic format (MGF) files were submitted to MASCOT v2.2 (Matrix Sciences Ltd, United Kingdom) for searching against a newly released RS barnacle larval protein dataset (92,117 target sequence entries that matched to 43,921 unique GI numbers), developed in-house from transcriptome data. The mass tolerance for the peptide was set to 10 ppm, and the MS/MS fragment ion tolerance was 0.5 Da. A maximum of one missed cleavage was allowed. Variable modifications were set to 4-plex iTRAQ and oxidation was set to methionine (M). The fixed modifications were set to methylethanethiosulfonate at cysteine and lysine, and 4-plex iTRAQ at N-terminal. The MASCOT files (.dat files) were analyzed using Scaffold v4.1.1 (Proteome Software Inc. USA) software for validation and quantitation of peptide and protein identifications. The peptide and protein identification threshold was set at 95 % using the Peptide and Protein Prophet algorithm with Scaffold delta-mass correction [63, 64]. Using these criteria the false positive rates for peptide and protein were 1 % and 0.1 %, respectively.

### Protein quantitation

iTRAQ label-based quantitation of the identified proteins was performed using the Scaffold Q+ algorithm. The intensities of all labeled peptides were normalized across all runs. Individual quantitative data acquired in each run were normalized using the i-Tracker algorithm, as described previously [65]. Peptide intensity was normalized within the assigned protein. The reference channel (e.g. 114) was normalized to produce a 1:1 fold change, and the iTRAQ ratios were then transformed to a log scale. *P* values were calculated using a paired *t*-test. All normalization calculations were performed as described previously [61].

### Statistical analysis

The sources of variation between biological and technical replicates of HK and RS samples were evaluated using unsupervised multivariate principal component analysis (PCA), using the Multiple Array Viewer (MeV) data analysis and visualization tool [66]. Log2-transformed fold changes from six replicates were used for the analysis. Sample selection was set to Cluster, and the centering mode was set to Median. A hierarchical cluster analysis for differentially-expressed proteins from replicates of HK and RS samples was performed using MeV. Tree selection was set to Gene Tree, sample was set to Tree, the Pearson correlation was set to distance matrix selection, average linkage clustering was set to linkage method selection, and centroid linkage was selected as the clustering method.

### Supporting data

The sequence datasets obtained during this project have been deposited in the NCBI BioProject ID: PRJNA256251 (http://www.ncbi.nlm.nih.gov/bioproject/256251).

## Additional files

Additional file 1: Table S1. Gene ontology (biological process) of assembled unigenes of newly released nauplii larvae of *B. amphitrite.* Biological process information was obtained by transferring homologous genes to barnacle contigs using a custom transcript. **Table S2.** Top 10 gene ontology terms for assembled unigenes of newly released nauplii larvae of *B. amphitrite*. Selected genes involved in a) larval development, b) signal transduction, c) in response to environment stressors, and d) osmoregulation. **Table S3.** Gene ontology (molecular function) for assembled unigenes of newly released nauplii larvae of *B. amphitrite*. Molecular process information was obtained by transferring homologous genes to barnacle contigs using a custom transcript. **Table S4.** Gene ontology (cellular component) for assembled contigs of newly released nauplii larvae of *B. amphitrite*. Cellular component information was obtained by transferring homologous genes to barnacle contigs using a custom transcript. (XLSX 57 kb) Additional file 2: Table S5. Top 40 abundant genes identified from the transcriptome of newly released nauplii larvae of *B. amphitrite*. (XLSX 14 kb) Additional file 3: Table S6. Top 30 Pfam domains predicted from the transcriptome of newly released nauplii of *B. amphitrite*. (XLSX 11 kb) Additional file 4: Table S7 The number of contigs linked to the Reactome pathway in newly released nauplii larvae of *B. amphitrite*. (XLSX 10 kb) Additional file 5: Table S8. Identified proteins and fold change ratios for Hong Kong and Red Sea larvae. (XLSX 168 kb) Additional file 6: Table S9. Up-regulated proteins involved in the stress response and osmoregulation in newly released nauplii larvae of Red Sea *B. amphitrite*. Hits were based on the number of unique peptides identified, the spectrum count and sequence coverage. (XLSX 24 kb) Additional file 7: Table S10. Down-regulated proteins involved in homeostasis and energy metabolism in newly released nauplii larvae of Red Sea *B. amphitrite*. Hits were based on the number of unique peptides identified, the spectrum count and sequence coverage. (XLSX 31 kb) Additional file 8: Table S11. List of differentially expressed proteins involved in cellular metabolism in newly released nauplii larvae of Red Sea *B. amphitrite*. Hits were based on the number of unique peptides identified, the spectrum count and sequence coverage. (XLSX 59 kb) Additional file 9: Table S12. Abundance of transcripts categorized to stress response, development, and signaling mechanisms, compared in parallel with protein regulation. (XLSX 9 kb) Additional file 10: Table S13. Environmental differences between the Red Sea and Hong Kong marine environments. (PDF 99 kb) Additional file 11: Figure S1. Newly-released nauplii of the Red Sea barnacle *B. amphitrite* used for transcriptome and proteome analyses. (PDF 56 kb)

## Abbreviations

AnxIXA: Annexin
BCS-2: Barnacle cyprid-specific protein 2
BP: Biological process
CA: Carbonic anhydrase
CC: Cellular component
CID: Collision-induced dissociation
Col4: Collagen alpha 4
CPNs: Chaperonins
CV: Coefficient variation
CyPA: Cyclophilin
EGFR15: Epidermal growth factor receptor
FABP: Fatty acid binding proteins
FDPase: Fructose 1,6-bisphosphatase
GNBPα: Guanine nucleotide-binding protein
GO: Gene ontology
GSTs: Glutathione S-transferases
HCD: Higher-energy collisional dissociation
HK: Hong Kong
HSP: Heat shock proteins
INX2: Innexin 2
LC: Liquid chromatography
MeV: Multiple array viewer
MGF: Mascot generic format
MGN: Major egg antigen
MIM: Mitochondrial inner membrane proteins
MP: Molecular function
NO: Nitric oxide
ORF: Open reading frame
PCA: Principal component analysis
RACK2: Receptor for protein kinase C
RanGTPBP1: GTP-binding nuclear protein
RS: Red Sea
SCX: Strong cation exchange chromatography
SelM: Selenoprotein
SLC2C: Phosphate transport protein
TCP1: T-complex protein
VDAC2: Voltage-dependent anion channel
α7-nAChR: Neuronal acetylcholine receptor
