# Supplementary material for: Transcriptome and proteome dynamics in larvae of the barnacle Balanus Amphitrite from the Red Sea
Source: BMC Genomics. 2015 Dec 15;16:1063. doi: 10.1186/s12864-015-2262-1 (PMC4678614; doi:10.1186/s12864-015-2262-1)
Supplement: Additional file 10: Table S13. — Environmental differences between the Red Sea and Hong Kong marine environments. (PDF 99 kb) [file 12864_2015_2262_MOESM10_ESM.pdf]

Additional file 10: Table S13. Environmental differences between Red Sea* and Hong Kong** marine environment. The measurements were taken from the locations where the sampling was conducted. (Hong Kong water measurement values are indicated in parentheses).

**Parameter**	**Location 1**	**Location 2**	**Location 3**	**Average**	**STDEV**
Salinity (psu)	42.14 (31.2)	42.59 (31.3)	42.22 (31.4)	42.31 (31.3)	0.24 (0.1)
Temperature (°C)	34.4 (29.5)	33.6 (29)	34.7 (29)	34.23 (29.16)	0.568 (0.288)
pH	8.2 (8.2)	8.17 (8.1)	8.23 (8.1)	8.2 (8.13)	0.03 (0.05)
Dissolved Oxygen (mg/L)	4.32 (7.3)	5.22 (6.2)	5.66 (6.1)	5.06 (6.53)	0.683 (0.665)

*King Abdullah University of Science and Technology (27°18′168″N, 35°05′661″E), Thuwal, Kingdom of Saudi Arabia

**Pak Sha Wan (22°21′45″N, 114°15′35″E), Water Control Zone, Hong Kong environmental protection department, Hong Kong.
