# Supplementary figures and images for: Transcriptome and proteome dynamics in larvae of the barnacle Balanus Amphitrite from the Red Sea
Source: BMC Genomics. 2015 Dec 15;16:1063. doi: 10.1186/s12864-015-2262-1 (PMC4678614; doi:10.1186/s12864-015-2262-1)

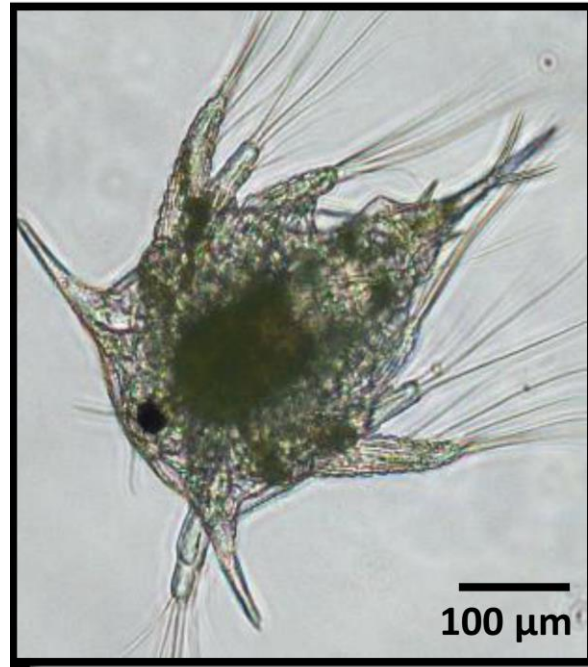

Supplement: Additional file 11: Figure S1. — Newly-released nauplii of the Red Sea barnacle B. amphitrite used for transcriptome and proteome analyses. (PDF 56 kb) [file 12864_2015_2262_MOESM11_ESM.pdf]
